# Editorial: Advanced imaging in breast cancer: New hopes, new horizons!

**DOI:** 10.3389/fonc.2023.1155500

**Published:** 2023-02-21

**Authors:** Abhishek Mahajan, Jinita Majithia

**Affiliations:** ^1^ The Clatterbridge Cancer Centre, Liverpool, United Kingdom; ^2^ Tata Memorial Hospital, Mumbai, India

**Keywords:** breast cancer, imaging, artificial intelligence, imaging biomarkers, functional imaging

Frontier’s in Oncology is a coveted journal encompassing technologies, fields and categories related to Cancer Research. An internationally recognized journal, Frontier’s in Oncology is cited by more than 25,000 articles over the past few years. Under the dedicated leadership and guidance of Dr. Abhishek Mahajan, Consultant Radiologist at Clatterbridge Cancer Centre, Liverpool, UK, and ex-Consultant Radiologist at Tata Memorial Hospital, Mumbai, India, having more than 15 years of experience in Oncoimaging, the Research Topic of “Advanced Imaging in Breast Cancer” hopes to present high quality articles with a goal to continuously strive towards advancing the knowledge and understanding of Breast Cancer.

## Introduction

Breast cancer is the most commonly diagnosed cancer worldwide with more than 2 million new cases in 2020. The incidence of breast cancer has increased over the past few decades and this can be attributed to change in the risk factor profiles, better cancer registration and advances in cancer detection. Breast cancer is the fifth most common cause of cancer-related deaths globally, with a disturbing estimated number of 2.3 million new cases, as per the GLOBOCAN 2020 data (1), and the global burden is only expected to increase. The breast cancer morbidity and mortality rates have significantly increased over the past few decades (2). There are more lost disability-adjusted life years (DALYs) by women to breast cancer globally than any other type of cancer. Breast cancer also represents a large social and economic burden to the society. The increase in the fiscal costs borne by the Government and Insurance companies as well as the emotional and physical costs borne by the patient and their families is unparalleled.

In order to maintain a good survival rate there have been constant efforts by scientists, researchers and the medical fraternity to not only introduce effective screening programs for early detection, but also for updating their knowledge database about evolving risk factors and epidemiology and recent advances in imaging for monitoring and surveillance of breast cancer in order to provide insights into new treatment strategies and patient stratifications that can impact management and eventual outcome of a patient with breast cancer.

Various new breast imaging tools have been identified and are being constantly developed to further improve our current ability to identify and treat early-stage breast cancer. This include advances in current technologies as well as introduction and implementation of new breast imaging platforms. Mammography is the gold standard first line imaging technique in preliminary diagnosis. Advances like Digital breast Tomosynthesis (DBT) as well as Volumetric Breast Density add incremental value in characterising normal and abnormal findings in the clinical setting of primary evaluation as well as post operative follow up in breast cancer (3). However, breast lesions identified on mammography usually require further investigation by ultrasonography. Ultrasonography (USG) has a useful role in characterizing the tumor as well as guiding interventions. There are recent advances in fusion based approach of mammography and ultrasonography, developing algorithms which improve the accuracy of computer-aided diagnosis (CAD) for analyzing breast lesion on ipsilateral mammography views (4). Recently, Contrast enhanced Mammography (CEM), a novel imaging modality, was developed as an adjunct to mammography to provide additional physiologic information about local breast perfusion in order to characterize enhancing lesions of the breast. Magnetic Resonance Imaging (MRI), however has been a game changer in breast imaging owing to its high soft tissue resolution and its use is becoming more prevalent, providing detailed three dimensional and cross-sectional models which are highly useful in diagnosis and monitoring. Diffusion weighted imaging (DWI) particularly has revolutionized oncological imaging, by giving vital qualitative and quantitative information regarding tumor biology that helps in detection, characterization and post treatment surveillance of lesions (5). Over the past years, Positron Emission Tomography (PET) has seen progress from being solely a research tool to replacing conventional imaging in various types of cancers (6). In breast imaging, there is strong supporting evidence that PET-CT is more rigorous than CT alone for revelation of breast lesions and distant metastases (7).

## Current challenges and opportunities in advanced breast imaging

1. Lack of awareness, delay in seeking healthcare, delay from the healthcare provider’s side, shortage of resources and high attrition rate have been few of the largest roadblocks. The emphasis of early diagnosis of breast cancer cannot be stressed upon enough in an era where there is evidence that newer emerging imaging techniques have facilitated improved outcome upon timely treatment. Beginning with education of women for self-breast examination to effective government policies for screening, one can ensure prompt diagnosis.
2. Requirement of large and homogeneous data representation across the globe has been another challenge. The varying demographics of breast cancer in different populations coupled with different socio-economic backgrounds and disorganized policies has not allowed for formulation and implementation of uniform and universally accepted protocols for screening programs. Encouraging and promoting incentives for research as well as providing support and funding from influential agencies and national and international health-care departments can positively impact and motivate the medical fraternity for continued research and development.
3. The controversies surrounding the appropriate techniques for imaging of breast cancer has been an on-going and ever-growing debate among radiologists. The acceptance of newer imaging modalities has been a serious challenge especially in developing nations, where the resistance towards use of these advanced techniques is not only because of the lack of awareness and training of the health care provider but also the lack of availability and funding. Acceptance and adequate penetration of newer health-care technologies can significantly improve the greater good of precision medicine.
4. Lack of expertise among health care providers regarding revolution in cancer research that has ushered in following the introduction of Artificial Intelligence (AI). There is an existing gap between the acceptance and implementation of AI in healthcare worldwide. Adoption of AI in clinical workflow applications can enable doctors and hospitals to offer new healthcare services. Government initiatives, ethical considerations and joint public private sector collaborations will ease the progress of AI, especially in developing countries (8).
5. Adding fuel to fire was the COVID pandemic. Besides the socio-economic and financial crisis to humankind, the pandemic also negatively impacted health-care by delaying research, which was on one of its fastest tracks in centuries (9). As the world continues to be at war and grapples with the aftermath of this disaster, health care providers and radiologists began realize the challenges of the large number of advanced breast cancer cases. There is always a silver lining though. The thrust towards setting up newer and more advanced health-care technologies came from the pressing need of the large number of backlog cases of breast cancer and also for providing remote health-care.

## Articles in the Research Topic

The aim of this Research Topic is to provide a diverse selection of stimulating research in the field of breast cancer by respected authors who have sacrificed countless hours in trying to bring forth well-researched and eloquent articles.This Research Topic aims to identify new ways researchers are refining and optimizing techniques in order to improve patient outcomes in diagnosed cases of breast cancer and to enable better monitoring and follow up throughout the course of treatment.

In this Research Topic, we present 21 topics, 16 of which are original research articles, 3 are systematic reviews and 2 are case reports.

The two unique case reports presented include one by Wei et al., who discuss a rare case of Inflammatory MyofibroblasticTumor (IMT) of the Breast with insights in the clinical, imaging and pathological findings. The other is by Jiang et al., discussing the imaging features (especially highlighting the MRI characteristics), pathology and clinical management of Mucocele-Like Tumor of the Breast Associated With Ductal Carcinoma In Situ.

One of the review articles, by Lei et al. discusses the challenges and future perspectives of the application of Artificial Intelligence in Medical Imaging of the Breast. A systemic review and meta-analysis, by Jiang et al., highlights the accuracy and feasibility of Sentinel Lymph Node Biopsy Mapped With Carbon Nanoparticle Suspensions in Patients With Breast Cancer. A systemic review article by Majithia et al., discuss the comprehensive literature on the various imaging appearances of fat necrosis in the breast.

Gao et al. conducted a study to assess the Screening Efficiency of Breast Cancer by Combining Conventional Medical Imaging Examinations With Circulating Tumor Cells.Amongst original research work done in the field of Artificial Intelligence, Zhang et al. discuss the clinical application of Ultrasound Image Deep Learning Model in Evaluating the Accuracy of Breast Cancer and Molecular Subtype Diagnosis. Song et al. evaluated the use of Texture Analysis, Using Semiquantitative Kinetic Parameter Maps from Dynamic Contrast Enhanced MRI, an imaging biomarker for pre-operative prediction of HER2 status in Breast Cancer. Similarly, Zhang et al. discuss the use of Texture Analysis of Dynamic Contrast Enhanced MRI Intra-tumoral Subregions to Identify Benign and Malignant Breast Tumors. A multi-centre prospective study by Zhao et al., elaborate on the enhancing diagnostic performance of breast ultrasound for patients with opportunistic screening-detected breast lesions by a Deep Learning-Based System.

Wang et al. evaluated the Combined Use of Shear Wave Elastography, Microvascular Doppler Ultrasound Technique and BI-RADS for the Differentiation of Benign and Malignant Breast Masses. With their retrospective multicentre study, Zhang et al., aimed at developing and validating and interpretable and simple-to-use ultrasound nomogram that is based on quantitative morphometric features for the prediction of breast malignancy. In a prospective study, Zhang et al., studied the association between vascular index (in Doppler evaluation) measured *via* Superb Microvascular Imaging and molecular subtype of invasive breast cancer and concluded that there was certain degree of correlation between the two and that vascular index has a limited role in predicting the luminal type A with high sensitivity and triple-negative subtype with high specificity. Zhao et al. investigated the diagnostic value of contrast-enhanced cone-beam breast computed tomography (CE-CBBCT) in predicting breast lesion with rim enhancement for malignancy. There were three studies on MRI, particularly centred around the use of Diffusion Weighted sequence. One was by He et al., exploring the Applications of Diffusion Weighted Imaging Techniques for Differentiating Benign and Malignant Breast Lesions. Another was a study by Lv et al. to evaluate the role of apparent diffusion coefficient (ADC) values obtained from diffusion-weighted imaging (DWI) in the differentiation of malignant from benign papillary breast lesions. A retrospective study by Yang et al. evaluated the performance of readout-segmented echo-planar imaging DWI (rs-EPI DWI) in detecting and characterizing breast cancers in a large Chinese cohort in comparison to dynamic contrast-enhanced MRI.

Bourgeois et al., evaluated the distribution of free indocyanine green following intravenous injection, in Near-Infrared Fluorescence imaging of breast cancer and axillary lymph nodes. Li et al. developed a comprehensive model for diagnosis and differentiation of primary breast lymphoma from breast cancer.

In a non-imaging based study, He et al. studied the Feasibility and Clinical Value of CT-Guided ^125^I Brachytherapy for Pain Palliation in Patients With Breast Cancer and Bone Metastases After External Beam Radiotherapy Failure. Another non-imaging based original article based on Animal model, Wang et al., envisioned that intraoperative real-time fluorescence imaging with a human serum albumin decorated indocyanine green probe could enable complete surgical removal of breast cancer in a mouse model.

## Conclusion

The field of breast imaging is always evolving with significant advances. There is always a learning curve in the interpretation of new technologies, and as medical practitioners we owe it to the vast global community to constantly advance ourselves in order to come up with more advanced technologies that can serve humanity. This will further aid the development of novel approaches for precision medicine in breast cancer. This issue aims to provide a comprehensive summary of recent advances and possibilities in the field of clinical breast imaging. The support of our esteemed authors, expertise of our panel of reviewers, round-the-clock hard work of the editorial and production staff and the keen interest of our beloved readers keeps the motivation going for Frontier’s in Oncology to continue to contribute to the ever-expanding field of breast oncology and to strive better each time.

## Author contributions

All authors listed have made a substantial, direct, and intellectual contribution to the work and approved it for publication.
